# Supplementary figures and images for: The impact of storage conditions on human stool 16S rRNA microbiome composition and diversity
Source: PeerJ. 2019 Dec 2;7:e8133. doi: 10.7717/peerj.8133 (PMC6894433; doi:10.7717/peerj.8133)

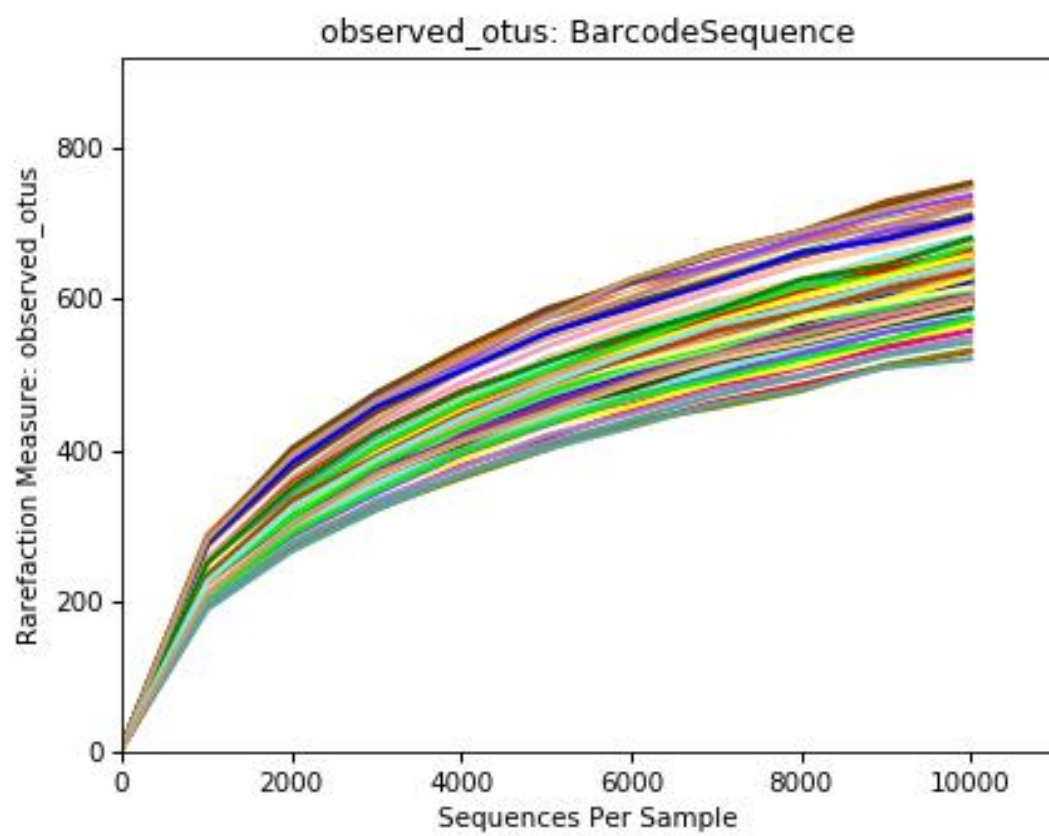

Supplement: Supplemental Information 1 [file peerj-07-8133-s001.pdf]

**A**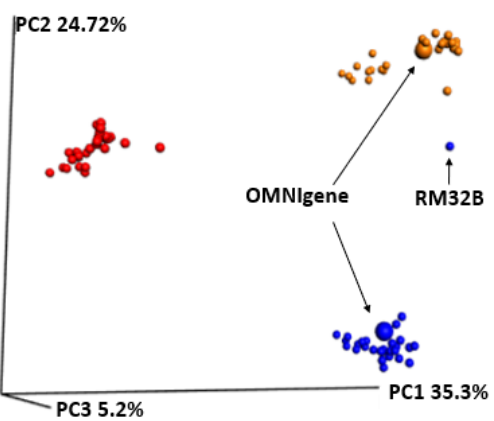**B**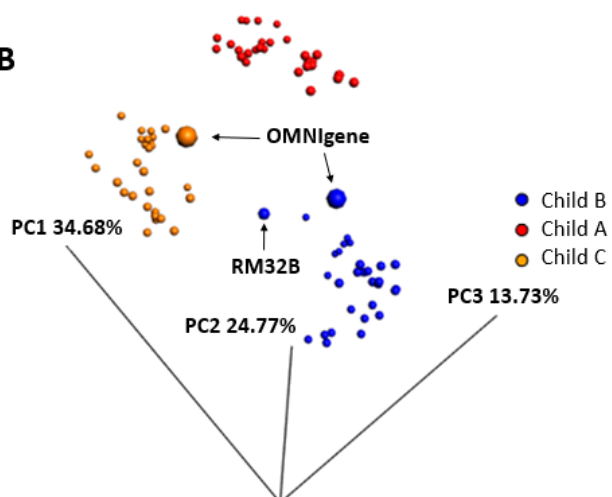

Supplement: Supplemental Information 2 [file peerj-07-8133-s002.pdf]

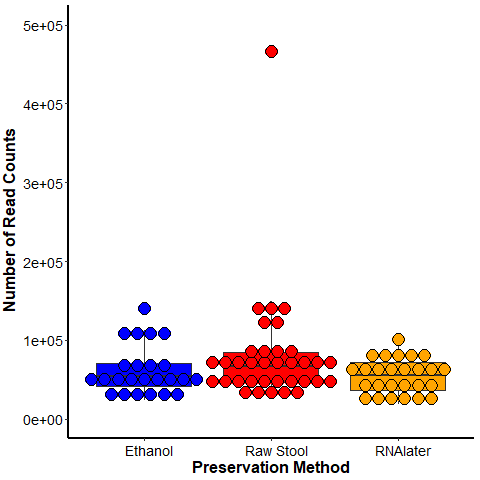

Supplement: Supplemental Information 3 — Number of read counts per sample across each preservation method used. [file peerj-07-8133-s003.png]

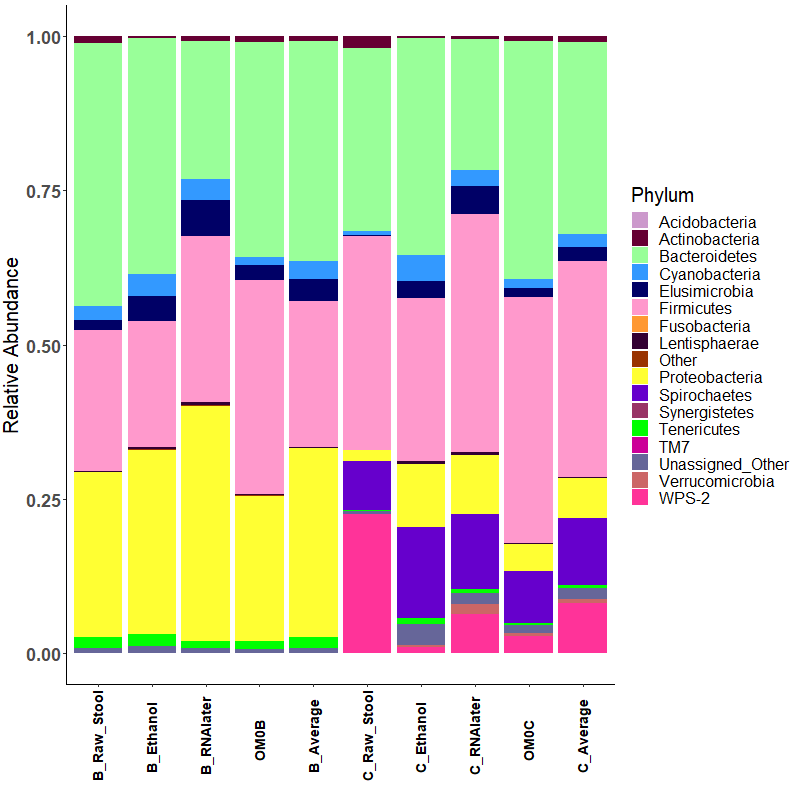

Supplement: Supplemental Information 4 — Child B (B_RNAlater, B_Raw_Stool, B_Ethanol and OM0B) and child C (C_RNAlater, C_Raw_Stool, C_Ethanol and OM0C). [file peerj-07-8133-s004.png]

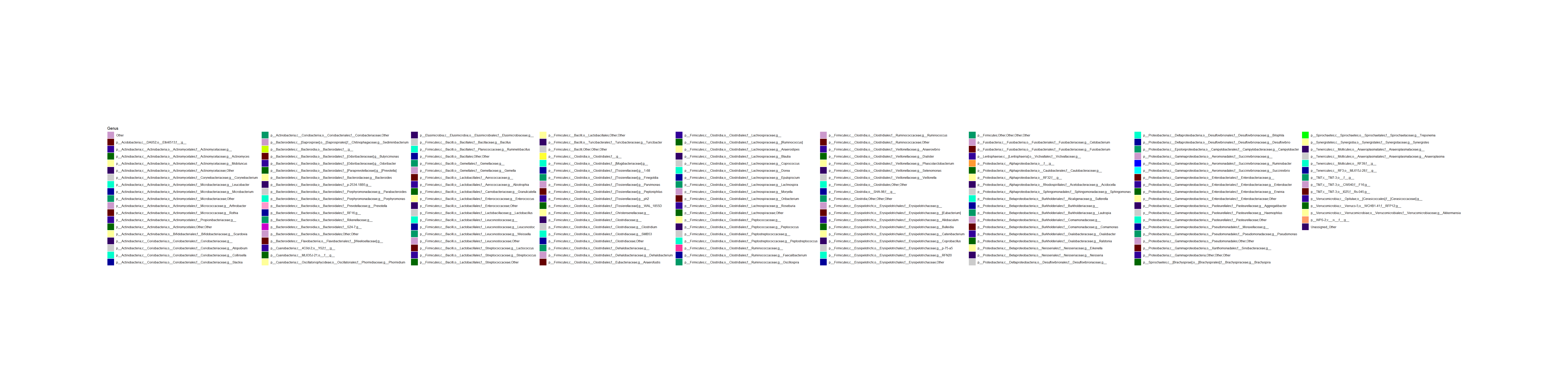

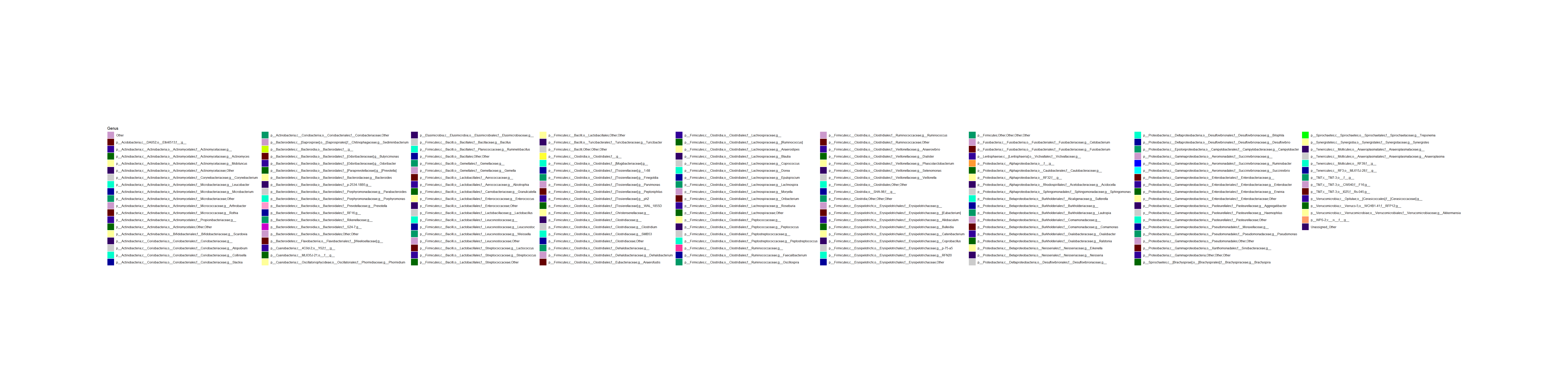

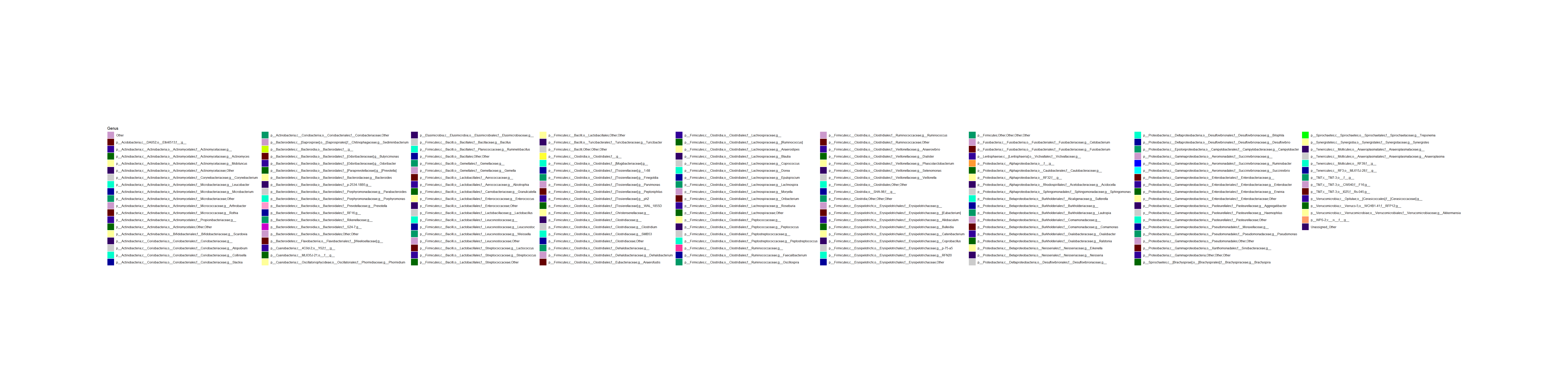

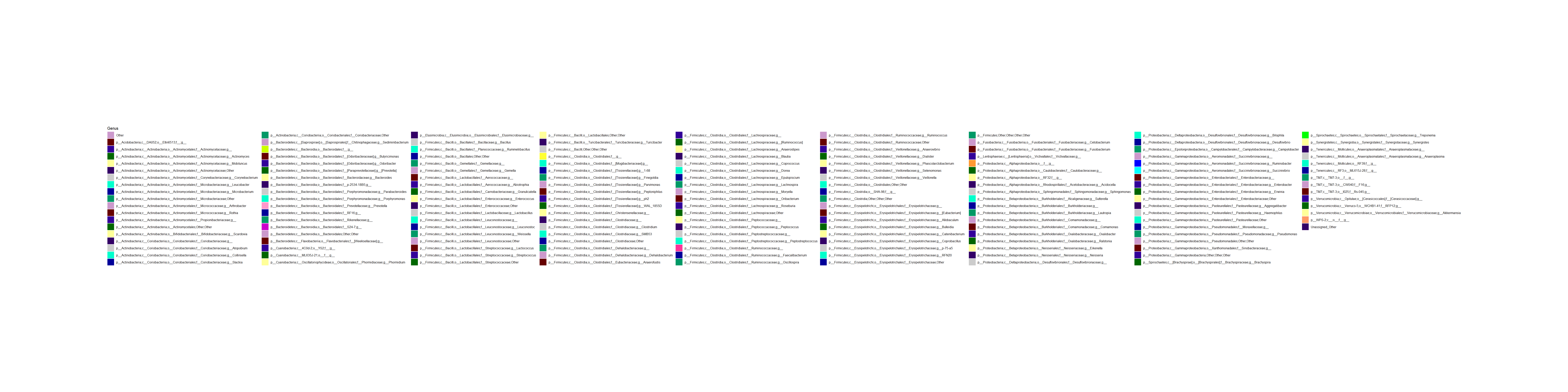

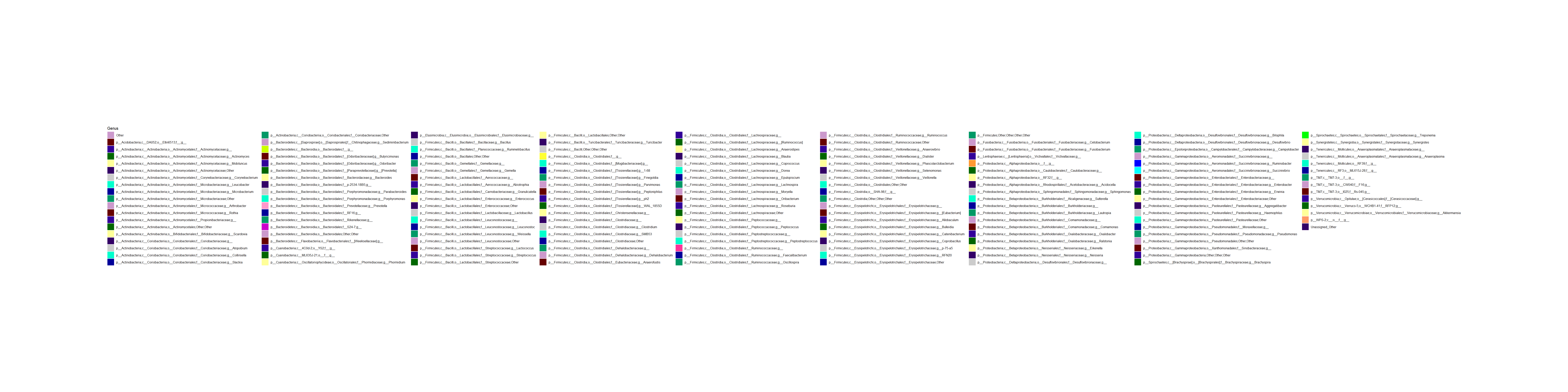

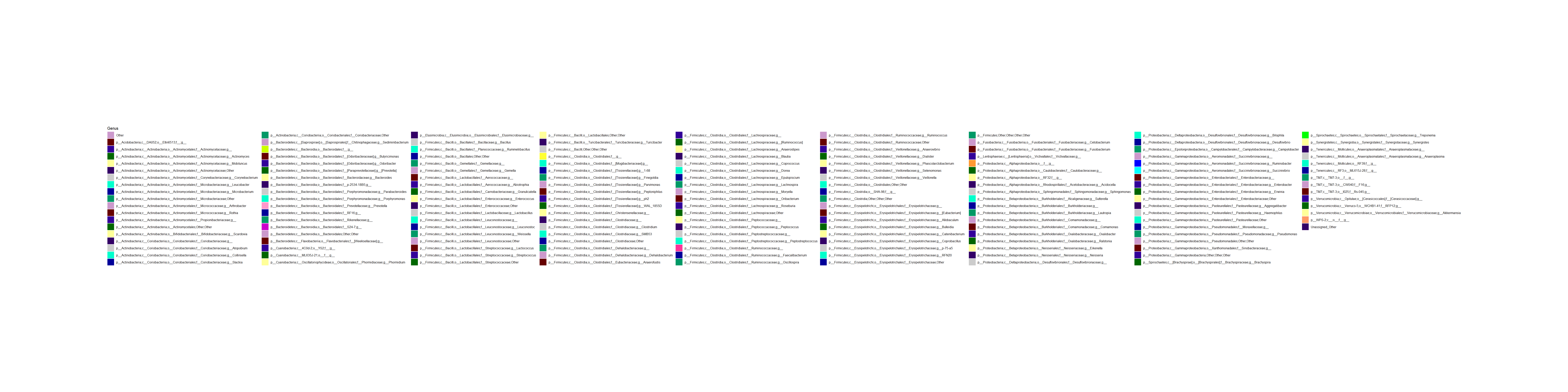

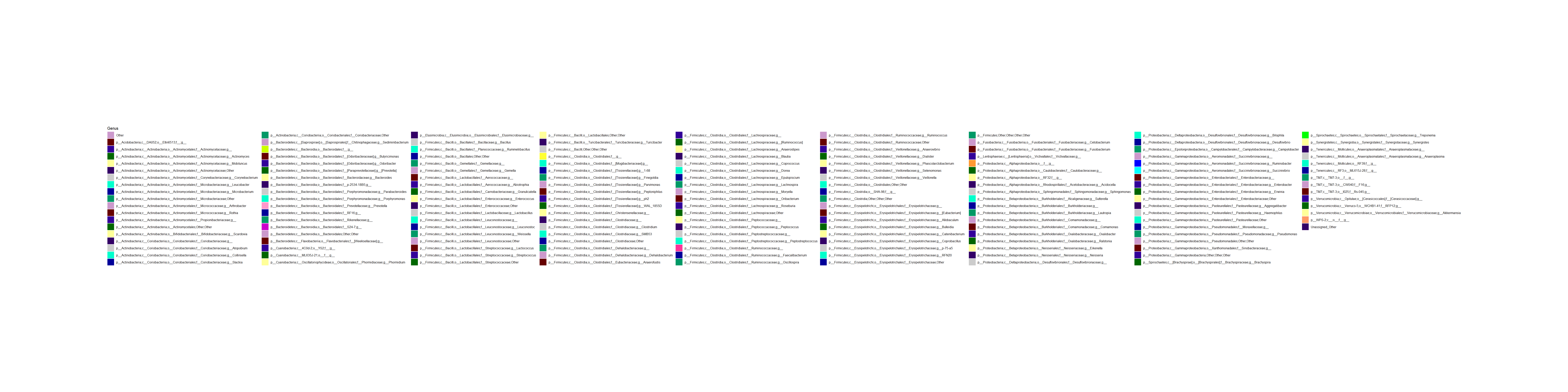

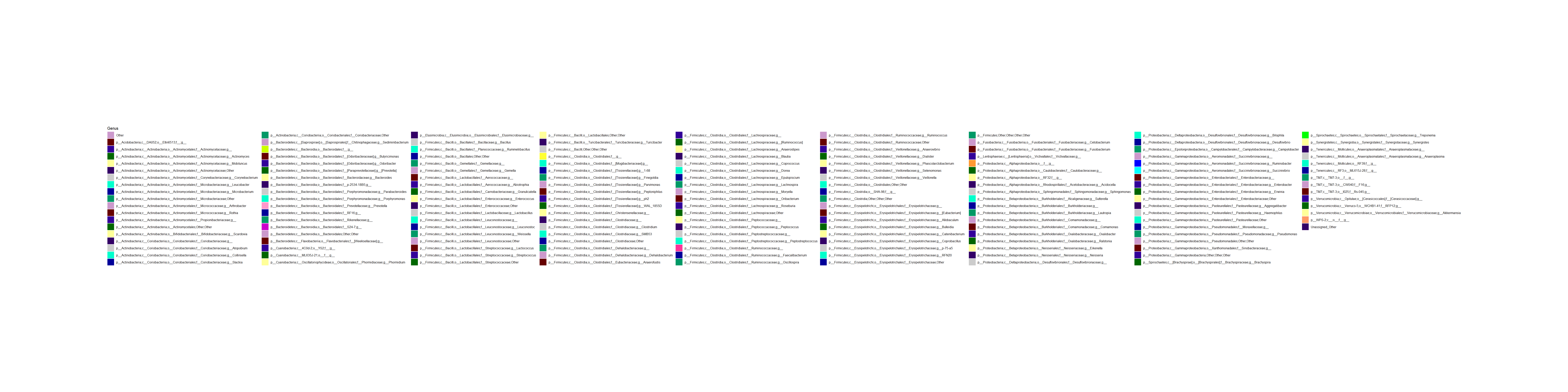

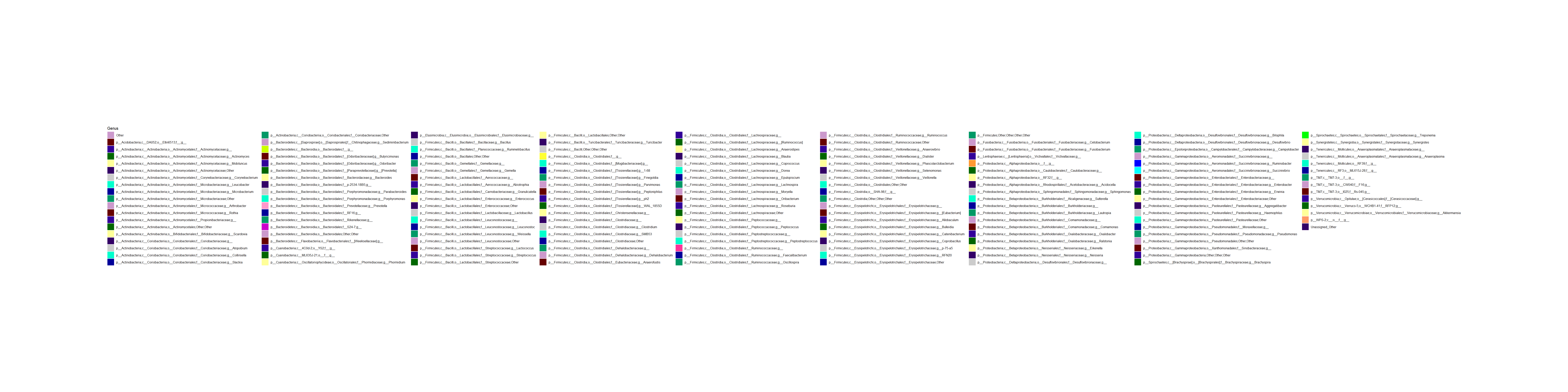

Supplement: Supplemental Information 11 — Full genus legend for Figs. 2B, 4D, 4E, 4F and 6B. Genera order listed goes from the top 1.00 (Other) on the relative abundance axis in alphabetical order downwards to 0 (Unassigned_Other). Colours are repeated every 10 colours. The average top 10 relatively abundant genera have be given 10 unique colours to make them more easily identifiable. [file peerj-07-8133-s011.docx]
