# Supplementary material for: The impact of storage conditions on human stool 16S rRNA microbiome composition and diversity
Source: PeerJ. 2019 Dec 2;7:e8133. doi: 10.7717/peerj.8133 (PMC6894433; doi:10.7717/peerj.8133)
Supplement: Supplemental Information 5 — Time zero was taken as the time at which all the stool samples, taken from an individual stool specimen, had been processed into all the collection tubes for the relevant conditions to be tested, which was approximately 30 min after defecation. Each sample was named using a code of four components: C/E/R/O (control, ethanol, RNAlater, OMNIgene.GUT) to indicate the preservation method, I/O/M to indicate the stool region (inner, outer, mixed), then a number indicating time-to-freezing (ranging from 0 to 32 h) followed by a letter indicating the stool donor (child A/B/C). An additional zero at the end of the code represents a replicate sample from the stool specimen. OMNIgene.GUT samples were stored at time 0 h but were not frozen prior to DNA extraction. Sample EM32C was excluded from further analysis due to low read count of 29 reads (Fig. S1), and sample RM32B was excluded from the analysis due to inconsistent child clustering by PCoA (Fig. S2) as it was suspected that the sample may have originated from or was contaminated with sample provided by a different individual. [file peerj-07-8133-s005.docx]

| Sample Name | Storage Method | Stool Region | Time-to-freezing (h) | Child |
| --- | --- | --- | --- | --- |
| CI0A | Raw Stool | Inner | 0 | A |
| CI0B | Raw Stool | Inner | 0 | B |
| CI0C | Raw Stool | Inner | 0 | C |
| CO0A | Raw Stool | Outer | 0 | A |
| CO0B | Raw Stool | Outer | 0 | B |
| CO0C | Raw Stool | Outer | 0 | C |
| CM0A | Raw Stool | Mixed | 0 | A |
| CM0A0 | Raw Stool | Mixed | 0 | A |
| CM1A | Raw Stool | Mixed | 1 | A |
| CM2A | Raw Stool | Mixed | 2 | A |
| CM4A | Raw Stool | Mixed | 4 | A |
| CM4A0 | Raw Stool | Mixed | 4 | A |
| CM8A | Raw Stool | Mixed | 8 | A |
| CM16A | Raw Stool | Mixed | 16 | A |
| CM16A0 | Raw Stool | Mixed | 16 | A |
| CM32A | Raw Stool | Mixed | 32 | A |
| CM0B | Raw Stool | Mixed | 0 | B |
| CM0B0 | Raw Stool | Mixed | 0 | B |
| CM1B | Raw Stool | Mixed | 1 | B |
| CM2B | Raw Stool | Mixed | 2 | B |
| CM4B | Raw Stool | Mixed | 4 | B |
| CM4B0 | Raw Stool | Mixed | 4 | B |
| CM8B | Raw Stool | Mixed | 8 | B |
| CM16B | Raw Stool | Mixed | 16 | B |
| CM16B0 | Raw Stool | Mixed | 16 | B |
| CM32B | Raw Stool | Mixed | 32 | B |
| CM0C | Raw Stool | Mixed | 0 | C |
| CM0C0 | Raw Stool | Mixed | 0 | C |
| CM1C | Raw Stool | Mixed | 1 | C |
| CM2C | Raw Stool | Mixed | 2 | C |
| CM4C | Raw Stool | Mixed | 4 | C |
| CM4C0 | Raw Stool | Mixed | 4 | C |
| CM8C | Raw Stool | Mixed | 8 | C |
| CM16C | Raw Stool | Mixed | 16 | C |
| CM32C | Raw Stool | Mixed | 32 | C |
| EI0B | Ethanol | Inner | 0 | B |
| EI0C | Ethanol | Inner | 0 | C |
| EO0B | Ethanol | Outer | 0 | B |
| EO0C | Ethanol | Outer | 0 | C |
| EM0A | Ethanol | Mixed | 0 | A |
| EM1A | Ethanol | Mixed | 1 | A |
| EM2A | Ethanol | Mixed | 2 | A |
| EM4A | Ethanol | Mixed | 4 | A |
| EM8A | Ethanol | Mixed | 8 | A |
| EM16A | Ethanol | Mixed | 16 | A |
| EM32A | Ethanol | Mixed | 32 | A |
| EM0B | Ethanol | Mixed | 0 | B |
| EM1B | Ethanol | Mixed | 1 | B |
| EM2B | Ethanol | Mixed | 2 | B |
| EM4B | Ethanol | Mixed | 4 | B |
| EM8B | Ethanol | Mixed | 8 | B |
| EM16B | Ethanol | Mixed | 16 | B |
| EM32B | Ethanol | Mixed | 32 | B |
| EM0C | Ethanol | Mixed | 0 | C |
| EM1C | Ethanol | Mixed | 1 | C |
| EM2C | Ethanol | Mixed | 2 | C |
| EM4C | Ethanol | Mixed | 4 | C |
| EM8C | Ethanol | Mixed | 8 | C |
| EM16C | Ethanol | Mixed | 16 | C |
| EM32C | Ethanol | Mixed | 32 | C |
| RI0A | RNAlater | Inner | 0 | A |
| RI0B | RNAlater | Inner | 0 | B |
| RI0C | RNAlater | Inner | 0 | C |
| RO0A | RNAlater | Outer | 0 | A |
| RO0B | RNAlater | Outer | 0 | B |
| RO0C | RNAlater | Outer | 0 | C |
| RM0A | RNAlater | Mixed | 0 | A |
| RM1A | RNAlater | Mixed | 1 | A |
| RM2A | RNAlater | Mixed | 2 | A |
| RM4A | RNAlater | Mixed | 4 | A |
| RM8A | RNAlater | Mixed | 8 | A |
| RM16A | RNAlater | Mixed | 16 | A |
| RM32A | RNAlater | Mixed | 32 | A |
| RM0B | RNAlater | Mixed | 0 | B |
| RM1B | RNAlater | Mixed | 1 | B |
| RM2B | RNAlater | Mixed | 2 | B |
| RM4B | RNAlater | Mixed | 4 | B |
| RM8B | RNAlater | Mixed | 8 | B |
| RM16B | RNAlater | Mixed | 16 | B |
| RM32B | RNAlater | Mixed | 32 | B |
| RM0C | RNAlater | Mixed | 0 | C |
| RM1C | RNAlater | Mixed | 1 | C |
| RM2C | RNAlater | Mixed | 2 | C |
| RM4C | RNAlater | Mixed | 4 | C |
| RM8C | RNAlater | Mixed | 8 | C |
| RM16C | RNAlater | Mixed | 16 | C |
| RM32C | RNAlater | Mixed | 32 | C |
| OM0B | OMNIgene.GUT | Mixed | NA, unfrozen | B |
| OM0C | OMNIgene.GUT | Mixed | NA, unfrozen | C |
