# Supplementary material for: The impact of storage conditions on human stool 16S rRNA microbiome composition and diversity
Source: PeerJ. 2019 Dec 2;7:e8133. doi: 10.7717/peerj.8133 (PMC6894433; doi:10.7717/peerj.8133)
Supplement: Supplemental Information 6 — Phyla were assigned using 97% clustering of the 16S rRNA gene to the Greengenes version 13.8 database (DeSantis et al., 2006) with Quantitative Insights Into Microbial Ecology (QIIME) software version 1.9.1 (Caporaso et al., 2010) in Python version 2.7. [file peerj-07-8133-s006.docx]

|  | Relative Abundance | | |
| --- | --- | --- | --- |
| Phylum Identification | Child A | Child B | Child C |
| Unassigned Other | 0.008737 | 0.009622 | 0.016543 |
| Other | 0 | 0.000683 | 6.24E-07 |
| Acidobacteria | 1.59E-06 | 0 | 1.25E-06 |
| Actinobacteria | 0.009748 | 0.007914 | 0.010671 |
| Bacteroidetes | 0.406698 | 0.369486 | 0.286562 |
| Cyanobacteria | 0.023088 | 0.029715 | 0.022466 |
| Elusimicrobia | 7.95E-07 | 0.035142 | 0.022826 |
| Firmicutes | 0.400726 | 0.226167 | 0.340702 |
| Fusobacteria | 1.59E-06 | 3.32E-05 | 9.98E-06 |
| Lentisphaerae | 0.000254 | 0.002178 | 0.002595 |
| Proteobacteria | 0.071497 | 0.301607 | 0.063646 |
| Spirochaetes | 0.072033 | 4.92E-06 | 0.106614 |
| Synergistetes | 0 | 0 | 2.37E-05 |
| TM7 | 3.98E-06 | 5.54E-06 | 9.36E-06 |
| Tenericutes | 0.007208 | 0.017426 | 0.004922 |
| Verrucomicrobia | 1.19E-06 | 0 | 0.006437 |
| WPS-2 | 1.59E-06 | 1.60E-05 | 0.115971 |
