# Supplementary material for: The impact of storage conditions on human stool 16S rRNA microbiome composition and diversity
Source: PeerJ. 2019 Dec 2;7:e8133. doi: 10.7717/peerj.8133 (PMC6894433; doi:10.7717/peerj.8133)
Supplement: Supplemental Information 10 — Alpha diversity scores were calculated for each sample using the core_diversity_analyses.py script in QIIME (Code S1) (Caporaso et al., 2010). [file peerj-07-8133-s010.docx]

A

|  | Mean Alpha Diversity Measure Score | | |
| --- | --- | --- | --- |
| Storage Method | Species Richness | Shannon | Simpson |
| Raw Control (35) | 635 | 99 | 31 |
| Ethanol (24) | 584 | 103 | 38 |
| RNAlater (26) | 664 | 111 | 35 |
| OMNIgene.GUT (2) | 726 | 154 | 60 |

B

|  | Alpha Diversity Measure Score Range | | |
| --- | --- | --- | --- |
| Storage Method | Species Richness | Shannon | Simpson |
| Raw Control (35) | 556 – 747 | 57 – 153 | 8 – 67 |
| Ethanol (24) | 526 – 649 | 69 – 141 | 22 – 62 |
| RNAlater (26) | 590 – 749 | 63 – 175 | 15 – 57 |
| OMNIgene.GUT (2) | 723 – 729 | 122 – 187 | 40 – 80 |

C

|  | Median Alpha Diversity Measure Score | | |
| --- | --- | --- | --- |
| Storage Method | Species Richness | Shannon | Simpson |
| Raw Control (35) | 636 | 99 | 27 |
| Ethanol (24) | 585 | 105 | 38 |
| RNAlater (26) | 651 | 107 | 37 |
| OMNIgene.GUT (2) | - | - | - |
