# Supplementary material for: The impact of storage conditions on human stool 16S rRNA microbiome composition and diversity
Source: PeerJ. 2019 Dec 2;7:e8133. doi: 10.7717/peerj.8133 (PMC6894433; doi:10.7717/peerj.8133)
Supplement: Supplemental Information 12 [file peerj-07-8133-s012.pdf]

Microbiome Analysis Code

(Python, QIIME, koeken, LEfSe)

Fastqc

Fastqc –outdir=FastQC *.gz > FastQC/fastqc.log

Or if already unzipped .fastq

Cutadapt is used for trimming the reads (Martin 2011):

-q minimum quality

-m minimum length

-o output file or directory R for Read 1

-p same as -o for Read 2

Then names of the input files R1 first then R2.

Note: If need to rename, name R1 and R2 the same filename for each set.

cutadapt -q 20 -m 250 -o location/name_R1_trimmed.fastq -p location/name_R2_trimmed.fastq name*R1_001.fastq name*R2_001.fastq

Pandaseq merges Read 1 and Read 2 together (Masella et al. 2012):

-f forward read (use trimmed files)

-r reverse read (use trimmed files)

-w write to output files (fasta file)

-p and -q adaptors from mapping files

(Adaptor p = AGMGTTYGATYMTGGCTCAG and adaptor q = CTGCCTCCCGTAGGAGT).

-g log file (extra part of the code pandaseandaseq.log 2>&1 → append to file and do not overwrite it)

pandaseq -f location/name_trimmed.fastq -r location/name_trimmed.fastq -w location/name_combined.fasta -p AGMGTTYGATYMTGGCTCAG -q CTGCCTCCCGTAGGAGT -g pandaseandaseq.log 2>&1

Sed is a piece of code that can be used to replace all blank lines with NNNN in a file:

‘s means search for all

g’ globally finds all not just the 1st

sed -i 's/^$/NNNN/g' location/filename

Grep is used to search for a term with a file:

* will search for all search terms within the given directory

Or location/filename instead of *

```
grep -A 5 -n "search term" location/*
```

Quantitative Insights Into Microbial Ecology (QIIME) (Caporaso et al. 2010):

Add QIIME labels:

-i location and name of input file (output of pandaseq)

-o location and name of output file

-m location and name of mapping file / metadata file

-c Description (column header from mapping file with fasta file names listed in it)

Add QIIME labels is combing all the pandaseq together into one big file adding on specific QIIME tags to each sample read.

```
add_qiime_labels.py -i combined/filename.fasta -o location/name_combined_seqs -m
location/mapping_filename.csv -c Description
```

Aligning to Greengenes database (DeSantis et al. 2006):

-i location and input filename (output of add_qiime_labels)

-o location and output filename

-p additional code (pick_closed_param.txt)

--suppress_step4 when you pass the piece of code step 4 of the code does not run (optional)

--min_otu_size value

```
pick_open_reference_otus.py -i location/input_file_name -o location/output_filename -p
pick_closed_param.txt --suppress_step4 --min_otu_size 5
```

(Default: PyNAST).

This is the piece of code contained in pick_closed_param.txt:

```
pick_otus:enable_rev_strand_match True
```

Core diversity analysis:

-i input file – input_biom_nofailures files produced after pick_open_reference_otus.py

-o output file (creates itself)

- m mapping file

-c categories to compare (column headings in mapping file)

-e sampling depth (maximum refraction depth to look at)

-t tree file produced after pick_open_reference_otus.py

-p can also pass a parameter file (for my work all_alpha_statistics)

core_diversity_analyses.py -i location/input_biom_nofailures_file -o location/output_filename -m location/mapping_filename -c "categories,time,treatment,group" -e 10000 -p all_alpha_statistics -t location/tree_filename.tre

This is the piece of code contained in all_alpha_statistics:

alpha_diversity:metrics observed_species,observed_otus,shannon,simpson,PD_whole_tree,chao1

To perform **LEfSe** (Segata et al. 2011) comparisons:

Koeken:

-i input_biom file

-o output folder (created automatically)

-m mapping file

-cl class from mapping file to compare (n=1)

-sp time series (set Time = 1 in all column rows if no time series)

koeken.py -i input_biom_file_from_OTUs -o output_folder -m mapping_file -cl class_name -sp Time

Data from koeken.py is then transferred into R Studio to make figures.

To filter samples from the OTU table:

-i input biom file

-o output file name of choice .biom

-m mapping file

-s ‘Sample_Group_of_interest_to_be_filtered:yes’

filter_samples_from_otu_table.py -i input_file_biom(nofailures) -o outputfile.biom -m mapping_file -s 'Treatment:yes'

Converting biom files to tsv to edit and then converting back to biom format:

```
biom convert --to-tsv --header -key taxonomy --output-metadata-id "ConsensusLineage"

biom convert -i otu_table.txt -o new_otu_table.biom --to-hdf5 --table-type="OTU table" --
processobs-metadata taxonomy
```

Note: Treatment, Time, Group and Category are all required as headings in the QIIME mapping file (also known as a metadata file).

Treatment = storage preservation method

Time = time-to-freezing

Category = stool region

Group = individual children

References

QIIME Scripts: http://qiime.org/scripts/

Caporaso JG, Kuczynski J, Stombaugh J, Bittinger K, Bushman FD, Costello EK, Fierer N, Peña AG, Goodrich JK, Gordon JI, Huttley GA, Kelley ST, Knights D, Koenig JE, Ley RE, Lozupone CA, McDonald D, Muegge BD, Pirrung M, Reeder J, Sevinsky JR, Turnbaugh PJ, Walters WA, Widmann J, Yatsunenko T, Zaneveld J, and Knight R. 2010. QIIME allows analysis of high-throughput community sequencing data. Nat Methods 7:335-336. 10.1038/nmeth.f.303

DeSantis TZ, Hugenholtz P, Larsen N, Rojas M, Brodie EL, Keller K, Huber T, Dalevi D, Hu P, and Andersen GL. 2006. Greengenes, a chimera-checked 16S rRNA gene database and workbench compatible with ARB. Appl Environ Microbiol 72:5069-5072. 10.1128/AEM.03006-05

Martin M. 2011. Cutadapt removes adapter sequences from high-throughput sequencing reads. EMBnetjournal 17:10-12. https://doi.org/10.14806/ej.17.1.200

Masella AP, Bartram AK, Truszkowski JM, Brown DG, and Neufeld JD. 2012. PANDAseq: paired-end assembler for illumina sequences. BMC Bioinformatics 13:31. 10.1186/1471-2105-13-31

Segata N, Izard J, Waldron L, Gevers D, Miropolsky L, Garrett WS, and Huttenhower C. 2011. Metagenomic biomarker discovery and explanation. Genome Biol 12:R60. 10.1186/gb-2011-12-6-r60
