# Supplementary material for: The impact of storage conditions on human stool 16S rRNA microbiome composition and diversity
Source: PeerJ. 2019 Dec 2;7:e8133. doi: 10.7717/peerj.8133 (PMC6894433; doi:10.7717/peerj.8133)
Supplement: Supplemental Information 13 [file peerj-07-8133-s013.pdf]

#####

Lauren V Carruthers

Pilot Study Paper Alpha Diversity Model LVC

R Studio Notebook

#####

Load in required libraries

```{r}
	library(ggplot2)
	library(lme4)
	library(DHARMa)
	library(RLRsim)
	library("blmeco")
```

Load in data for all alpha diversity models and call it Data

```{r}
Data <- read.csv("FILE_LOCATION/FILENAME.csv", header = TRUE, row.names = 1)
head(Data)
```

There are 4 explanatory variables and a random effect variable (child)

# - category # (= stool region)

# - treatment # (= stool storage preservation method)

# - time # (= time-to-freezing of stool considered as a continuous variable)

# - as.factor(time) # (= time-to-freezing of stool as a factor)

cateogry was only compared at one time point therefore category time interactions are not included here.

Therefore the following interaction to consider are:

# - treatment:cateogry

# - treatment:time

# - treatment:as.factor(time)

# : only test method:time for example.

* will test method:time, method and time.

Note that time (continuous) and as.factor(time) (not continuous) cannot be tested in the same model so run two separate models to see which is best.

Set child as a factor

```{r}
Data$child <- as.factor(Data$child)
```

Include child as a random effect, only 3 children in this study.

Shannon models s

Richness models r

Simpson models p

Using backward elimination to create the models

####

Shannon Model Code

Create a null model with just children as a random effect

```{r}
s0 <- lmer(shannon ~ 1 + (1|child), data = Data)
summary(s0)
```

Now create full model s1 using continuous time

```{r}
s1 <- lmer(shannon ~ treatment + time + category + treatment:time + treatment:category +
(1|child), data = Data)
summary(s1)
```

Compare s0 and s1:

```{r}
anova(s0, s1, test = "Chisq")
```

s1 is not significantly different to s0 so retain s0 (p > 0.05)

None of the model components are significant predictors of shannon diversity, double check by backward elimination. s0 best fit model.

Create model s2 removing treatment:category

```{r}
s2 <- lmer(shannon ~ treatment + time + category + treatment:time + (1|child), data = Data)
summary(s2)
```

Compare model s2 with model s0

````
```{r}
anova (s0,s2, test = "Chisq")
```
# s2 is not significantly different to s0 so retain s0 (p > 0.05)


# Create model s3 removing  treatment:time
```{r}
s3 <- lmer(shannon ~ treatment + time + category +  (1|child), data = Data)
summary(s3)
```
# Compare model s3 with s0
```{r}
anova(s0,s3,test="Chisq")
```
# s3 is not significantly different to s0 so retain s0 (p > 0.05)



# Create model s4 removing category
```{r}
s4 <- lmer(shannon ~ treatment + time + (1|child), data = Data)
summary(s4)
```
# Compare model s0 and s4
```{r}
anova(s0,s4,test="Chisq")
```
# s4 is not significantly different to s0 so retain s0 (p > 0.05)



# Create model s5 removing time
```{r}
````
````

s5 <- lmer(shannon ~ treatment + (1|child), data = Data)

summary(s5)

```

# Compare model s0 and s5

```{r}

anova(s0,s5,test="Chisq")

```

s5 is not significantly different to s0 so retain s0 (p > 0.05)

#Therefore s0 is the best fit model here.

Now create full model s6 using as.factor(time)

```{r}

s6 <-lmer(shannon ~ treatment + as.factor(time)+ category + as.factor(time):treatment + treatment:category + (1|child), data= Data)

summary(s6)

```

Compare model s0 and s6

```{r}

anova(s0,s6,test="Chisq")

```

s6 is not significantly different to s0 so retain s0 (p > 0.05)

#Create model s7 removing treatment:category

```{r}

s7 <- lmer(shannon ~ treatment + as.factor(time)+ category + as.factor(time):treatment + (1|child), data= Data)

summary(s7)

```
```

Compare model s0 and s7

```{r}
anova(s0,s7,test="Chisq")
```

s7 is not significantly different to s0 so retain s0 (p > 0.05)

Create model s8 removing as.factor(time):treatment

```{r}
s8 <- lmer(shannon ~ treatment + as.factor(time)+ category + (1|child), data= Data)
summary(s8)
```

Compare model s0 and s8

```{r}
anova(s0,s8,test="Chisq")
```

s8 is not significantly different to s0 so retain s0 (p > 0.05)

Create model s9 removing as.factor(time)

```{r}
s9 <- lmer(shannon ~ treatment + category + (1|child), data= Data)
summary(s9)
```

Compare model s0 and s9

```{r}
anova(s0,s9,test="Chisq")
```

s9 is not significantly different to s0 so retain s0 (p > 0.05)

s0 <- lmer(shannon ~ 1 + (1|child), data = Data) is the best fit model here.

Check the residuals of the model s0 are normally distributed

```{r}
hist(residuals(s0))
```

Compare the residuals with the fitted values

```{r}
plot(s0,type=c("p","smooth"))
```

Perform a qqnorm plot to evaluate the assumption of the normality of the residuals

```{r}
residuals_shannon =simulateResiduals(s0)
```

```{r}
testUniformity(simulationOutput = residuals_shannon)
```

Check the assumption of equal variance

```{r}
	plot(s0, sqrt(abs(resid(.)))~fitted(.), type = c("p", "smooth"), ylab= expression
(sqrt(abs(resid))))
```

time (continuous or as a factor), category and treatment are not important model components for shannon diversity prediction

####

Richness Model Code

Create a null model with just children as a random effect

```{r}

r0 <- lmer(richness ~ 1 + (1|child), data = Data)

summary(s0)

```

Now create full model r1 using continuous time

```{r}

r1 <- lmer(richness ~ treatment + time + category + treatment:time + treatment:category + (1|child), data = Data)

summary(r1)

```

Compare r0 and s1:

```{r}

anova(r0, r1, test = "Chisq")

```

r1 is significantly different to r0 so retain r1 (p < 0.05)

Create model r2 removing treatment:category

```{r}

r2 <- lmer(richness ~ treatment + time + category + treatment:time + (1|child), data = Data)

summary(r2)

```

Compare model r2 with model r1

```{r}

anova (r1,r2, test = "Chisq")

```

r2 is not significantly different to r1 so retain r2 (p > 0.05)

Create model r3 removing treatment:time

```{r}
```

````
r3 <- lmer(richness ~ treatment + time + category +  (1|child), data = Data)

summary(r3)

```

# Compare model r3 with r2

```{r}

anova(r2,r3,test="Chisq")

```

# r3 is not significantly different to r2 so retain r3 (p > 0.05)



# Create model r4 removing category

```{r}

r4 <- lmer(richness ~ treatment + time + (1|child), data = Data)

summary(r4)

```

# Compare model r3 and r4

```{r}

anova(r3,r4,test="Chisq")

```

# r4 is not significantly different to r3 so retain r4 (p > 0.05)



# Create model r5 removing time

```{r}

r5 <- lmer(richness ~ treatment +  (1|child), data = Data)

summary(r5)

```

# Compare model r4 and r5

```{r}

anova(r4,r5,test="Chisq")

```
````
````

````
# r5 is not significantly different to r4 so retain r5 (p > 0.05)

# Compare model r0 and r5

```{r}

anova(r0,r5,test="Chisq")

```

# r5 is significantly different to r0 so retain r5 (p < 0.05)

# Now create full model r6 using as.factor(time)

```{r}

r6 <-lmer(richness ~ treatment + as.factor(time)+ category + as.factor(time):treatment +
treatment:category + (1|child), data= Data)

summary(r6)

```

# Compare model r0 and r6

```{r}

anova(r0,r6,test="Chisq")

```

# r6 is significantly different to r0 so retain r6 (p < 0.05)

#Create model r7 removing treatment:category

```{r}

r7 <- lmer(shannon ~ treatment + as.factor(time)+ category + as.factor(time):treatment + (1|child),
data= Data)

summary(r7)

```

# Compare model r0 and r7

```{r}

anova(r0,r7,test="Chisq")
````
````

~~~
```
# r7 is not significantly different to r6 so retain r6 (p > 0.05)


# Create model r8 removing as.factor(time):treatment
```{r}
r8 <- lmer(richness ~ treatment + as.factor(time)+ category + (1|child), data= Data)
summary(r8)
```
# Compare model r7 and r8
```{r}
anova(r7,r8,test="Chisq")
```
# r8 is not significantly different to r7 so retain r8 (p > 0.05)


# Create model r9 removing as.factor(time)
```{r}
r9 <- lmer(richness ~ treatment + category + (1|child), data= Data)
summary(r9)
```
# Compare model r8 and r9
```{r}
anova(r8,r9,test="Chisq")
```
# r9 is not significantly different to r8 so retain r8 (p > 0.05)

# r9 = r3 therefore r5 is the best fit model here


# r5 <- lmer(richness ~ treatment + (1|child), data = Data) is the best fit model here.
~~~

Check the residuals of the model r5 are normally distributed

```{r}
hist(residuals(r5))
```

Compare the residuals with the fitted values

```{r}
plot(r5,type=c("p","smooth"))
```

Perform a qqnorm plot to evaluate the assumption of the normality of the residuals

```{r}
residuals_richness =simulateResiduals(r5)
```

```{r}
testUniformity(simulationOutput = residuals_richness)
```

Check the assumption of equal variance

```{r}
plot(r5, sqrt(abs(resid(.)))~fitted(.), type = c("p", "smooth"), ylab= expression (sqrt(abs(resid))))
```

time (continuous or as a factor)and category are not important model components for species richness diversity prediction

####

Simpson Model Code

Create a null model with just children as a random effect

```{r}
p0 <- lmer(simpson ~ 1 + (1|child), data = Data)

summary(p0)
```

Now create full model s1 using continuous time

```{r}
p1 <- lmer(simpson ~ treatment + time + category + treatment:time + treatment:category + (1|child), data = Data)

summary(p1)
```

Compare p0 and p1:

```{r}
anova(p0, p1, test = "Chisq")
```

p1 is not significantly different to p0 so retain p0 (p > 0.05)

None of the model components are significant predictors of simpson diversity, double check by backward elimination. p0 best fit model.

Create model p2 removing treatment:category

```{r}
p2 <- lmer(simpson ~ treatment + time + category + treatment:time + (1|child), data = Data)

summary(p2)
```

Compare model p2 with model p0

```{r}
anova (p0,p2, test = "Chisq")
```

p2 is significantly different to p0 so retain p0 (p < 0.05)

# p = 0.04884 on balance point of significance

Create model p3 removing treatment:time

````
```{r}
p3 <- lmer(simpson ~ treatment + time + category +  (1|child), data = Data)
summary(p3)
```
# Compare model p3 with p2
```{r}
anova(p2,p3,test="Chisq")
```
# p3 is not significantly different to p2 so retain p3 (p > 0.05)



# Create model p4 removing category
```{r}
p4 <- lmer(simpson ~ treatment + time + (1|child), data = Data)
summary(p4)
```
# Compare model p3 and p4
```{r}
anova(p3,p4,test="Chisq")
```
# p4 is not significantly different to p3 so retain p4 (p > 0.05)

# Create model p5 removing time
```{r}
p5 <- lmer(simpson ~ treatment +  (1|child), data = Data)
summary(p5)
```
# Compare model p4 and p5
```{r}
anova(p4,p5,test="Chisq")
```
````

p5 is not significantly different to p4 so retain p5 (p > 0.05)

Compare p5 to the null model

```{r}
anova(p0,p5,test="Chisq")
```

p5 is not significantly different to p0 so retain p0 (p > 0.05)

Therefore p0 is considered the best fit model here.

```{r}
anova(p0,p2,test="Chisq")
```

```{r}
anova(p0,p3,test="Chisq")
```

```{r}
anova(p0,p4,test="Chisq")
```

Whilst models p2, p3 and p4 are statistically significant (assuming significant is p < 0.05) when compared to then null model, all p values here are very close to p = 0.05 suggesting all predictors here are borderline for significance and not important in the context of the paper questions.

Now create full model p6 using as.factor(time)

```{r}
p6 <-lmer(simpson ~ treatment + as.factor(time)+ category + as.factor(time):treatment + treatment:category + (1|child), data= Data)
summary(p6)
```

Compare model p5 and p6

```{r}
anova(p0,p6,test="Chisq")
```

p6 is not significantly different to p0 so retain p0 (p > 0.05)

#Create model p7 removing treatment:category

```{r}

p7 <- lmer(simpson ~ treatment + as.factor(time)+ category + as.factor(time):treatment + (1|child), data= Data)

summary(p7)

```

Compare model p0 and p7

```{r}

anova(p0,p7,test="Chisq")

```

p7 is not significantly different to p0 so retain p0 (p > 0.05)

Create model p8 removing as.factor(time):treatment

```{r}

p8 <- lmer(simpson ~ treatment + as.factor(time)+ category + (1|child), data= Data)

summary(p8)

```

Compare model p0 and p8

```{r}

anova(p0,p8,test="Chisq")

```

p8 is not significantly different to p0 so retain p0 (p > 0.05)

Create model p9 removing as.factor(time)

```{r}

p9 <- lmer(simpson ~ treatment + category + (1|child), data= Data)

summary(p9)
```

```

# Compare model p0 and p9

```{r}
anova(p0,p9,test="Chisq")
```

p9 is not significantly different to p0 so retain p0 (p > 0.05)

p0 <- lmer(simpson ~ 1 + (1|child), data = Data) is the best fit model here.

Check the residuals of the model p0 are normally distributed

```{r}
hist(residuals(p0))
```

Compare the residuals with the fitted values

```{r}
plot(p0,type=c("p","smooth"))
```

Perform a qqnorm plot to evaluate the assumption of the normality of the residuals

```{r}
residuals_simpson =simulateResiduals(p0)
```

```{r}
testUniformity(simulationOutput = residuals_simpson)
```

Check the assumption of equal variance

```{r}
plot(p0, sqrt(abs(resid(.)))~fitted(.), type = c("p", "smooth"), ylab= expression (sqrt(abs(resid))))
```
```

# time (continuous or as a factor), category and treatment are not important model components for simpson diversity prediction

####
